# Stable isotope labeling confirms mixotrophic nature of streamer biofilm communities at alkaline hot springs

**DOI:** 10.3389/fmicb.2015.00042

**Published:** 2015-02-05

**Authors:** Florence Schubotz, Lindsay E. Hays, D'Arcy R. Meyer-Dombard, Aimee Gillespie, Everett L. Shock, Roger E. Summons

**Affiliations:** ^1^Department of Earth, Atmospheric and Planetary Sciences, Massachusetts Institute of TechnologyCambridge, MA, USA; ^2^Department of Earth and Environmental Sciences, University of Illinois at ChicagoChicago, IL, USA; ^3^School of Earth and Planetary Sciences, Arizona State UniversityTempe, AZ, USA; ^4^Department of Chemistry and Biochemistry, Arizona State UniversityTempe, AZ, USA

**Keywords:** hot springs, streamer biofilm communities, stable isotope probing, Archaea, heterotrophy, Aquificae, Yellowstone National Park

## Abstract

Streamer biofilm communities (SBC) are often observed within chemosynthetic zones of Yellowstone hot spring outflow channels, where temperatures exceed those conducive to photosynthesis. Nearest the hydrothermal source (75–88°C) SBC comprise thermophilic Archaea and Bacteria, often mixed communities including Desulfurococcales and uncultured Crenarchaeota, as well as Aquificae and Thermus, each carrying diagnostic membrane lipid biomarkers. We tested the hypothesis that SBC can alternate their metabolism between autotrophy and heterotrophy depending on substrate availability. Feeding experiments were performed at two alkaline hot springs in Yellowstone National Park: Octopus Spring and “Bison Pool,” using various ^13^C-labeled substrates (bicarbonate, formate, acetate, and glucose) to determine the relative uptake of these different carbon sources. Highest ^13^C uptake, at both sites, was from acetate into almost all bacterial fatty acids, particularly into methyl-branched C_15_, C_17_ and C_19_ fatty acids that are diagnostic for Thermus/Meiothermus, and some Firmicutes as well as into universally common C_16:0_ and C_18:0_ fatty acids. ^13^C-glucose showed a similar, but a 10–30 times lower uptake across most fatty acids. ^13^C-bicarbonate uptake, signifying the presence of autotrophic communities was only significant at “Bison Pool” and was observed predominantly in non-specific saturated C_16_, C_18_, C_20_, and C_22_ fatty acids. Incorporation of ^13^C-formate occurred only at very low rates at “Bison Pool” and was almost undetectable at Octopus Spring, suggesting that formate is not an important carbon source for SBC. ^13^C-uptake into archaeal lipids occurred predominantly with ^13^C-acetate, suggesting also that archaeal communities at both springs have primarily heterotrophic carbon assimilation pathways. We hypothesize that these communities are energy-limited and predominantly nurtured by input of exogenous organic material, with only a small fraction being sustained by autotrophic growth.

## Introduction

Several alkaline hot springs of the Lower Geyser Basin at Yellowstone National Park are home to streamer biofilm communities (SBCs) that inhabit the chemosynthetic zones of the hot spring outflow channel. Such apparently prolific biomass formation in non-photosynthetic ecosystems invites questions regarding how these communities harvest carbon and energy for their sustainment. The microbial community composition of the SBCs can vary among different hot springs (Meyer-Dombard et al., 2011; Schubotz et al., 2013), but is usually dominated by bacterial thermophiles of the order Aquificae, Thermus/Meiothermus, and sometimes Thermotogae and thermophilic Crenarchaea of the order Desulfurococcales and Thermoproteales (Reysenbach et al., 1994; Meyer-Dombard et al., 2011; Swingley et al., 2012; Schubotz et al., 2013). SBCs are not found within all alkaline hot spring outflow channels and it has been hypothesized that easy access to nutrients and exogenous carbon sources that are washed into the pool might facilitate the growth of these communities (Swingley et al., 2012; Schubotz et al., 2013). SBCs are typically found associated with siliceous sinter deposits that have been precipitated from the hydrothermal fluids, but are also observed to colonize other material such as twigs, pine needles or bison excrement within the outflow channels thereby fostering the idea that these might serve as potential carbon sources for the streamer communities. Concomitantly, lipid biomarker studies reveal that SBCs collected from topographically elevated sinter deposits that experience limited exogenous input of organic material contain stable carbon isotopic signatures indicative of autotrophic carbon fixation, while hot springs situated closer to the elevations of a meadow (“Bison Pool”) or at the foot of a hill (Octopus Spring) appear to harbor either heterotrophic or mixotrophic biofilm communities (Schubotz et al., 2013). Notably, these differences in carbon metabolism are observed in lipids diagnostic for Aquificae, Thermus/Meiothermus, and Firmicutes suggesting that these groups of organisms can switch their metabolism depending on carbon availability. Indeed, a number of heterotrophic thermophiles have been isolated from Yellowstone hot springs (Zeikus et al., 1979; Huber et al., 1998; Schaffer, 2004; Johnson et al., 2006; Boyd et al., 2007; Osburn and Amend, 2010) and thermodynamic calculations have shown that small organic compounds present in the hydrothermal fluid, such as formate, could be significant sources of energy for chemosynthetic thermophiles (Shock et al., 2005; Windman et al., 2007; Shock et al., 2010). The current study was conducted to experimentally document main modes of carbon assimilation pathways of SBCs using compound-specific stable isotope probing.

Stable isotope labeling experiments in combination with lipid biomarker analysis allows the direct study of the activities of different groups of microorganisms if diagnostic biomarkers are present (Boschker and Middelburg, 2002). Fortunately, SBCs contain distinctive lipid biosignatures that can be traced to different thermophilic bacterial and archaeal organisms: Long-chain fatty acids such as C_20:1_ and *cy*-C_21:0_ were identified to be diagnostic lipids of the Aquificae (Jahnke et al., 2001), and are found in their intact form attached to aminopentanetetrol (APT) and phosphatidylinositol (PI) diacylglycerols (Sturt et al., 2004). Similarly, *iso* and *anteiso* branched C_15_, C_16_, C_17_, C_18_, and C_19_ fatty acids are signature lipids of Thermus/Meiothermus if they are found attached to diglycosyl(N-acetyl)glycosaminyl (NAcG-2G) diacylglycerols (Ferreira et al., 1999) or phospho(N-acetyl)glycosaminyl (NAcG-P) diacylglycerols (Yang et al., 2006), which each have an additional 2-hydroxyacyl group N-linked to the glycosamine. Crenarchaeal lipids of SBCs are dominated by glycosidic lipids with glycerol dibiphytanyl glycerol tetraether (GDGT) core structures having up to four pentacyclic rings (Schubotz et al., 2013). Overall there is a close agreement between intact polar membrane lipid analyses and phylogeny-based approaches (Meyer-Dombard et al., 2011; Swingley et al., 2012; Schubotz et al., 2013), indicating that membrane lipids in combination with stable isotope probing can be used to determine carbon sources for distinct microbial communities in SBCs.

Clues about the carbon assimilation pathways of SBCs were derived from natural abundance stable isotopic compositions of carbon compounds from various alkaline hot springs (Schubotz et al., 2013). In this study we compare two of these hydrothermal ecosystems, “Bison Pool” and Octopus Spring that seemingly harbor similar SBCs albeit with different modes of carbon assimilation. For instance at “Bison Pool,” Aquificae-diagnostic lipids often have heavy δ^13^*C*-values of −5–5‰. These isotopic compositions, which are close to those of dissolved inorganic carbon (DIC), have been assigned to autotrophic growth of Aquificae using the reverse tricarboxylic acid (TCA) cycle for carbon fixation, in accordance with cultured Aquificae representative *Thermocrinis ruber* where biomass was depleted in ^13^C by only 3.3‰ relative to the carbon source (Jahnke et al., 2001). Conversely, at Octopus Spring Aquificae-lipids were found to have light δ^13^*C*-values of −27 to −24‰, close to that of the dissolved organic carbon (DOC), suggestive of heterotrophic carbon assimilation. *Thermocrinis ruber* can indeed grow on formate as sole carbon source, resulting in similar isotopic fractionations as observed for Octopus Spring (Jahnke et al., 2001). These observations have led to the hypothesis that Aquificae in hot springs can alternate their carbon metabolism dependent on availability of organic carbon. To test the role of autotrophy versus heterotrophy in these systems we designed stable isotope probing experiments using ^13^C-labeled bicarbonate, acetate, formate and glucose as substrates for SBC growth under conditions as close as possible to *in situ* conditions.

Understanding hydrothermal ecosystems can provide insight into the types of environments that may have been habitable on the early Earth, and serve as models for possible extreme environments beyond our planet. Additionally, understanding how modern organisms live, and even thrive, using different metabolic pathways from those in more benign environments, can inform our study of the potential of life to inhabit a range of niches, on this planet and on others, and lead to a better understanding of the evolutionary history of these processes. This study aims to address how a community, living at a high-temperature edge of the chemosynthetic zone in hot springs, utilizes carbon and energy from its environment—two of the most fundamental requirements for living organisms.

## Materials and methods

### Sample collection and fluid geochemistry

Collections of SBCs were made in the upper temperature reaches of the outflow channels of “Bison Pool” (UTM, Zone 12: latitude 0510717, longitude 4935158) and Octopus Spring (UTM, Zone 12: latitude 0516053, longitude 4931215) in the Lower Geyser Basin of Yellowstone National Park in the summer field season of 2006, 2007 and 2009. Sample locations and Thermal Inventory ID Numbers (TIN) are: “Bison Pool” (TIN LSMG013) and Octopus Spring (TIN LXCG138). Temperature and pH were measured at each site prior to sample collection and are reported in Table 1. Concentrations and stable carbon isotopic composition of DIC, DOC, and total organic carbon (TOC) of streamer biofilms have been determined elsewhere (Havig et al., 2011; Meyer-Dombard et al., 2011; Swingley et al., 2012; Schubotz et al., 2013). Concentrations of acetate and formate were determined in 2006 at “Bison Pool” and Octopus Spring (Windman et al., 2007; Windman, 2010) and were assumed to be representative of values for the later samples.

**Table 1:** **Fluid geochemistry, bulk stable carbon isotope composition and concentrations of different carbon pools at the location of streamer biofilm community growth in the outflow channels of “Bison Pool” and Octopus Spring (data from Windman et al., 2007; Havig, 2009; Havig et al., 2011; Meyer-Dombard et al., 2011; and Schubotz et al., 2013)**.

	**Temp. (°C)**	**pH**	**DIC ppm C**	**δ^**13**^C (‰)**	**DOC ppm C**	**δ^**13**^C (‰)**	**TOC δ^**13**^C (‰)**	**Acetate ppm**	**Formate ppm**	**Dissolved O_**2**_ ppm**	**Cl-ppm**	**Total sulfide ppb**
**“BISON POOL”**												
2006	81.1	7.83	63.4 ± 0.5	0.16 ± 0.06	1.15 ± 0.05	−21.31	−7.1 ± 0.13	0.27^*^	0.36^*^	0.8	219.1 ± 0.1	85
2007	83.5	8.06	66.0 ± 0.7	−0.16 ± 0.21	1.0 ± 0.04	−22.92	*nd*	*nd*	*nd*	1.6	239.6 ± 0.9	59
2009	80.4	7.64	66.7 ± 1.3	0.48 ± 0.20	0.23 ± 0.02	−16.7	−17.8	*nd*	*nd*	0.3	233.8 ± 0.2	70
**OCTOPUS SPRING**												
2006	82.7	7.78	61.0 ± 2.1	−1.11 ± 0.18	1.01 ± 0.05	−25.97	−16.81 ± 0.13	bdl^*^	0.02^*^	1.7	241.4 ± 0.1	14
2007	80.9	7.79	63.0 ± 0.3	−1.87 ± 0.32	1.43 ± 0.04	−24.68	−17.9	*nd*	*nd*	0.7	245.4 ± 0.1	*bdl*
2009	85.4	7.90	60.0 ± 1.8	−0.83 ± 0.20	0.23 ± 0.02	*nd*	−17.4	*nd*	*nd*	0.3	200.9 ± 0.6	14

nd, no data; bdl, below detection limit (<25 ppb).

* These samples were collected further down the outflow channels: 71.1°C and pH 8.1 at “Bison Pool” and 67.5°C and pH 8.1 at Octopus Spring.

### Set-up of stable isotope probing experiments

Three types of stable isotope probing experiments were carried out over the course of this investigation: (1) Batch-fed incubations that were open to the atmosphere (in 2006 at “Bison Pool” and Octopus Spring), (2) Batch-fed incubations that were amended with H_2_ as electron donor (in 2007 at “Bison Pool”) and (3) Flow-through experiments using hydrothermal fluid directly from the hot spring (in 2009 at “Bison Pool” and Octopus Spring) (**Figure 2**). The third set of experiments most closely mirrored natural conditions.

#### Batch-fed incubations without amendment of electron donor

SBCs were collected in 2006 with solvent-cleaned forceps at “Bison Pool” and Octopus Spring and placed into Whirl-Pak sample bags that were amended with 50 mL hydrothermal fluid collected from the source of the hot spring. Each Whirl–Pak was inoculated with 99% ^13^C-labeled bicarbonate, formate or acetate (Cambridge Isotope Laboratories, Foster City, CA), with final concentrations (and % ^13^C-label assuming concentrations reported in Table 1) of 5 ppm (11% ^13^C), 4 ppm (92% ^13^C) 2 ppm (98% ^13^C), respectively. The Whirl–Pak bags were left open to allow exchange with the atmosphere and were placed in the outflow stream to incubate at *in situ* temperatures of ca. 80°C for 1, 4, 25, and 93 h at “Bison Pool” (only time points 1 and 93 h are analyzed in this study) and for 3, 20, and 67 h at Octopus Spring. Experiments were terminated and SBCs were killed with 0.025 M HgCl_2_ additions and frozen until further analysis.

#### Batch-fed incubations with amendment of H_2_

In 2007, SBCs collected in the outflow of “Bison Pool” were placed into 175 mL serum vials, filled with 70 mL of hydrothermal fluid from the source and inoculated with 99% ^13^C-labeled bicarbonate, formate or acetate, with final concentrations (and % ^13^C-label assuming concentrations reported in Table 1) of 250 ppm (79% ^13^C), 1 ppm (83% ^13^C) and 1.5 ppm (78% ^13^C), respectively, and were closed air-tight with butyl stoppers and amended with ca. 20% H_2_ in air at atmospheric pressure. The closed incubators were left in the outflow to incubate at *in situ* temperatures of ca. 84°C for 2 and 25 h. Field control samples were treated in the same way but had no addition of labeled substrate. Experiments were terminated and SBCs were killed with 0.025 M HgCl_2_ additions and frozen until further analysis.

#### Flow-through reactor incubations

**Figure 1:**
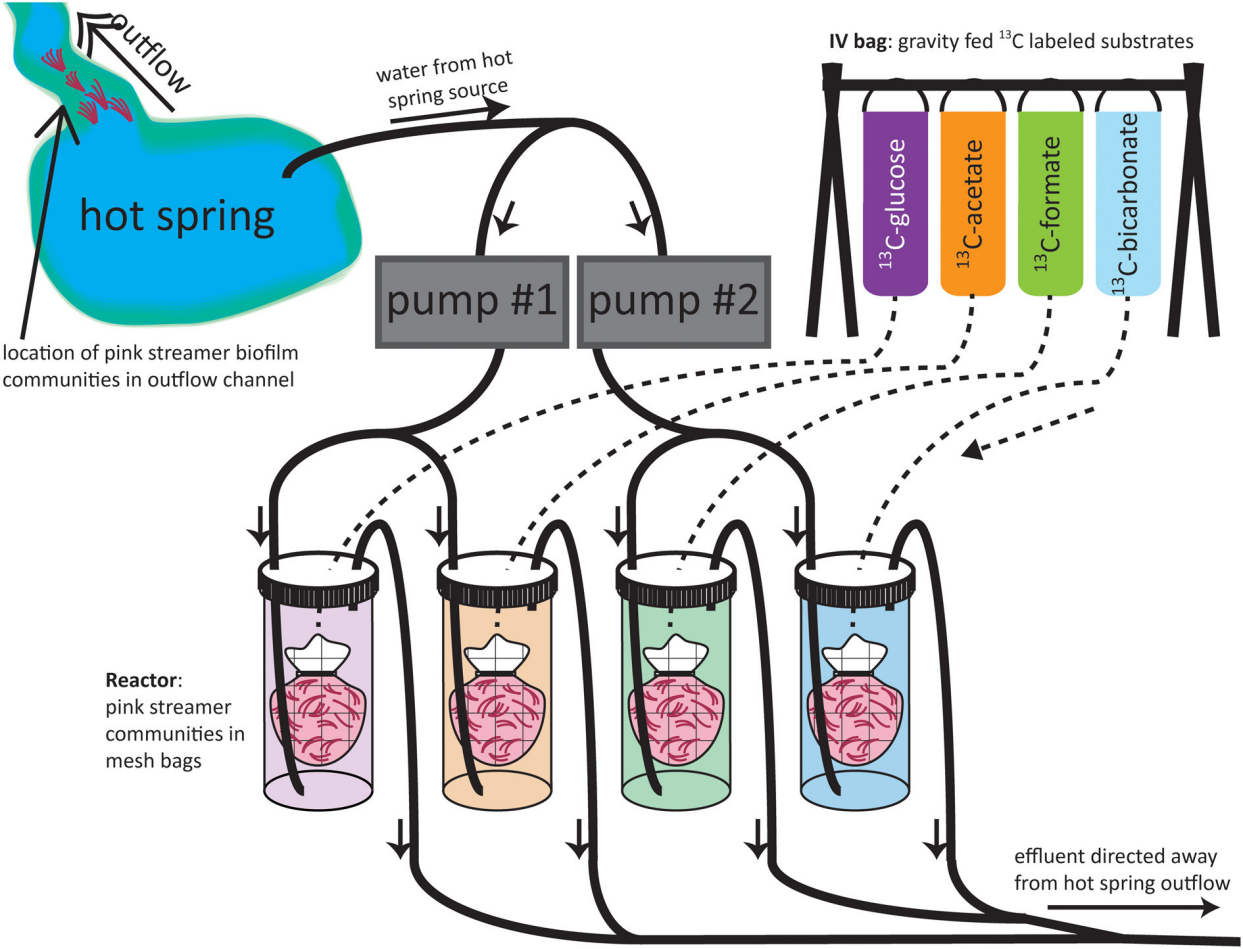
**Experimental set-up of flow-through reactors**. Solid lines indicate tubing carrying hot spring water; dashed lines indicate IV-tubing carrying labeled substrates. Arrows indicate direction of flow within the tubing.

In 2009, flow-through reactor systems were constructed to simulate improved *in situ* conditions for the incubation of SBCs by ensuring a constant inflow of an energy source provided by the hydrothermal fluid (Figure 1). For each experiment, SBCs were collected with forceps from the outflow channels of “Bison Pool” or Octopus Spring, homogenized, and then divided into four portions that were placed in mesh bags. Each of the four mesh bags containing streamer material was placed in one of four 1 L Nalgene bottle incubators. The incubator bottles were placed in the meadow next to the hot spring and each one was set up with plastic tubing (inner diameter 0.5 cm) connected to a pump for hot spring in-flow, separate tubing for outflow, and a rubber stopper. Two pumps were used, each attached to two incubators, drawing water from the hot spring with the two inflow source tubes placed as close to one another as possible in the hot spring source pool. The pumps were run continuously during the experiment, at a constant flow rate (ca. 660 mL/min), and were turned off only when collecting samples for the first and the final time points. Although temperatures were not monitored in the Nalgene bottles during the course of the experiment, the short length of the tubing from the hot spring to the bottles (<5 m), combined with the fast flow rate relative to the size of the tubing and the incubator bottles kept the experimental setup close to the spring outflow temperatures for the duration of SBC incubations. The labeled substrates were each added to individual intravenous (IV) drip-bags, along with 1 L of hot spring source water, and the IV-tubing connected these bags to the appropriate bottles (see Figure 1 for experimental set-up). The outflow from each incubator was directed away from the hot spring feature toward the surrounding meadow. Concentrations of 99% ^13^C-labeled substrates in the IV bags were 735 μM for bicarbonate, 906 μM for formate, 744 μM for acetate and 336 μM for glucose. With an IV-bag flow-rate of 1 drop/s equaling to approximately 3 mL/min and a pump flow rate of ca. 660 mL/min the substrate concentration in the incubators stabilized after 8 min, resulting in final substrate concentrations (and % ^13^C-label assuming substrate concentrations according to Table 1) of 1.1 mM (1.4% ^13^C) for bicarbonate, 12.1 μM (40% ^13^C) for formate, 8.1 μM (41% ^13^C) for acetate and 2.1 μM (73% ^13^C) for glucose. Concentrations for glucose were not determined but we assumed maximum concentrations of glucose to be one tenth of DOC (Table 1). In total three time points were collected: t0—without any addition of label, t1—incubated for 1 h and t2—incubated for 4 h at “Bison Pool” and 1.5 h at Octopus Spring. All samples were frozen at −20°C after the end of the experiment and stored until further analysis.

### Lipid extraction, separation, and analyses

Between 0.2 and 0.5 g of lyophilized biomass was ground to a fine powder and extracted with a modified Bligh and Dyer method (White and Ringelberg, 1998) as described previously (Schubotz et al., 2013) using a solvent mixture of methanol:dichloromethane:water, 10:5:4 for the first two extraction steps and methanol:dichloromethane:1% trichloroacetic acid in water, 10:5:4 in a third step. Supernatants were pooled and subjected to liquid-liquid extractions using 5 mL of dicholoromethane and water. The organic layer were collected as the total lipid extracts and dried under a gentle stream of N_2_ until further analysis.

#### High performance liquid chromatography-mass spectrometry (HPLC-MS) and preparative HPLC

An aliquot of the untreated total lipid extract was analyzed on an Agilent 1200 series HPLC system coupled to an Agilent 6520 Accurate-Mass Quadrupole Time-of-Flight Mass Spectrometer with an electrospray ionization interface. Intact polar membrane lipids were separated on a Waters Acquity UPLC BEH Amide column (3.5 μm, 2.1 × 150 mm) equipped with a guard column of the same packing material (Wörmer et al., 2013). Identification occurred via MS-MS experiments and exact masses (Schubotz et al., 2013), quantification of compound classes was accomplished by comparison of peak areas without taking into account differences in response factors due to the lack of authentic standards (Schubotz et al., 2009). Preparative HPLC was performed on samples from the 2009 stable isotope-probing experiments that showed little uptake of ^13^C into tetraether lipid-derived biphytanes. Here, the total lipid extract was separated into a polar fraction that contained intact polar tetraether lipids and an apolar fraction that contained tetraether lipids without polar headgroups. Separation of polar and apolar fractions was achieved on an InertSil Diol column (5 μm, 10 × 150 mm) with an Agilent 1200 series HPLC system coupled to a fraction collector and an Agilent 6130 single quadrupole to check for compound retention times, following the protocol described in Zhu et al. (2013).

#### Gas chromatography (GC)

Elemental sulfur was removed from the total lipid extracts using activated copper prior to gas chromatographic analyses. A 20% aliquot of the total lipid extract was transmethylated with 2.5% methanolic HCl (3 h at 70°C) yielding fatty acid methyl esters (FAMEs), alcohols, and ether lipids. The acid-hydrolyzed lipid extract was then separated on silica-gel-packed Pasteur pipette columns into five fractions using hexane (F1), hexane:dichloromethane, 8:2 (F2), dichloromethane (F3), dichloromethane:ethyl acetate, 8:2 (F4) and dichloromethane:methanol, 7:3 (F5). F3 contained the FAMEs, which were analyzed directly, F4 contained bacterial glycerol diethers (DEG) and archaeol, which were derivatized with *N,O*-bis(trimethylsilyl)trifluoro-acetamide in pyridine at 70°C for 1 h prior to GC analysis. 30% of the total lipid extracts from the 2006 and 2007 experiments and 50% of the polar fraction (yielded by preparative HPLC) of the 2009 experiments were subjected to boron-tribromide (BBr3) ether-cleavage treatment in order to liberate the hydrocarbon cores of archaeal and bacterial ether-bound lipids (Jahn et al., 2004). FAMEs, diether lipids and ether-cleavage products were identified on an Agilent 7890 GC coupled to an Agilent 5975C mass selective detector equipped with a Agilent J&W DB-1 fused silica capillary column (60 m, 0.32 mm × 0.25 μm). Quantification of individual compounds occurred after addition of known amounts of squalene standard to each fraction and subsequent analysis on an Agilent 7890 GC, equipped with the same column as the GC-MSD and a flame ionization detector (FID), as described previously (Schubotz et al., 2013). All lipid amounts were normalized to the dry weight of extracted biomass in gram (g).

#### GC-isotope ratio mass spectrometry

Compound-specific stable carbon isotopic compositions were determined with a Thermo TraceGC coupled to a ThermoFinnigan Deltaplus XL isotope ratio monitoring mass spectrometer via a combustion interface operated at 850°C. Column type and GC temperature program were identical to GC-FID and MS analyses. Stable carbon isotope ratios were determined relative to an external CO_2_ standard that was calibrated relative to a reference mixture of of *n*-alkanes (Mixture B) provided by Arndt Schimmelmann of Indiana University. Reported values for the isotopic compositions of lipids were corrected by mass balance for the carbon present in the trimethyl silyl (TMS) or methyl derivative where applicable. Standard deviations were determined by triplicate analysis. Carbon isotopic values are reported in the delta notation as δ^13^C relative to the Vienna PeeDee Belemnite (VPDB) Standard.

The carbon isotopic compositions and relative abundances of individual lipids were used to calculate the weighted mean (wm) of the isotopic composition of both archaeal and bacterial lipids as follows:

(1) $$\delta^{13}C_{wm}=\sum_{i\;=\;1}^{n}\left(\frac{peakarea_{lipid\;X}}{peakarea_{sum\;lipids}}\right)*\delta^{13}C_{lipid\;X}$$

Where peakarea*_lipidX_* is the peakarea of the individual lipid, δ^13^C*_lipidX_* the isotopic composition of the same lipid and peakarea*_sumlipids_* the summed peakareas of the measured lipids, i.e., bacterial FAMEs or archaeal ether lipids.

### Calculation of ^13^C uptake into lipids

Incorporation of ^13^C into lipid X can be expressed as the specific uptake, which is the excess ^13^C above background samples (equation 2) or more quantitatively as the total uptake of ^13^C (Middelburg et al., 2000). Total ^13^C uptake into lipid X (ng g^−1^) at a given time point t_*n*_ was determined as the product of excess ^13^C and concentration of the respective lipid,(equation 3), where the excess ^13^C is calculated as the difference between the fractional abundance ^13^F of the lipid at t_*n*_ and ^13^F at time-zero t_0_ (equation 4).

(2) $$\Delta \delta^{13}C\;=\delta^{13}{\text{C}}_{\text{sample}}-\delta^{13}{\text{C}}_{\text{control}}$$

(3) $${}^{13}\text{C uptake into lipid X }{\text{t}}_{\text{n}}\;={(}^{13}{\text{F}}_{\text{tn}}{-}^{13}{\text{F}}_{\text{t}0}\;)*\text{ conc}.\text{ lipid t}$$

(4) $$\begin{array}{l} \;{\;}^{13}\text{F }{=}^{13}\text{C}/{(}^{13}\text{C }{+}^{12}\text{C})\;=\text{R}/\left(\text{R}+1\right)\\ \text{ and R }=\left(\delta^{13}\text{C}/1000\;+1\right)\text{ x }{\text{R}}_{\text{VPDB}} \end{array}$$

Total carbon uptake rates (ng C g^−1^ day^−1^) were calculated using equation 5, where ^13^F was determined after equation 4, with ^13^C and ^12^C calculated as the sum of ^13^C or ^12^C in IV bag and hot spring multiplied by their respective flow rates (equation 6).

(5) $$\begin{array}{l} \text{Total carbon uptake rate into lipid X}{=}^{13}\text{​​C uptake into lipid /}\\ \;{\;}^{13}{\text{F}}_{\text{substrate in bioreactor}}\text{/duration of experiment} \end{array}$$

(6) $$\begin{array}{l} {}^{13}{\text{C}}_{\text{substrate in bioreactor}}=\text{ conc}.^{13}{\text{C}}_{\text{IV bag}}*\text{flow }{\text{rate}}_{\text{IV bag}}\\ \;+\text{ conc}.^{13}{\text{C}}_{\text{hot spring}}*\text{flow }{\text{rate}}_{\text{pump}} \end{array}$$

Errors of equation 2 were determined as the sum of the analytical standard deviations (determined by triplicate measurements) of δ^13^C_sample_ and δ^13^C_control_. Errors of equation 5 were determined through Gaussian error propagation of the analytical standard deviation (determined by triplicate measurements) of the individual lipid compounds.

## Results

### *In situ* geochemistry and membrane lipid composition

The hydrothermal fluids collected at the *in situ* location of SBC formation share similar geochemistry at “Bison Pool” and Octopus Spring over the summer years 2006–2009 (Table 1; Schubotz et al., 2013). Temperatures were between 80 and 85°C with pH 7.6–8.0. DIC concentrations ranged from 60 to 66 ppm, while DOC concentrations showed greater shifts ranging from 0.2 to 1.4 ppm. Stable carbon isotopic compositions (δ^13^C) for DIC also showed little variation, ranging from −1.8 to 0.5‰ for DIC, while of DOC had δ^13^*C*-values of −24.7 to −16.7‰ and was generally lighter at Octopus Spring. δ^13^C of TOC was similar at both sites over the years with −17.8 to −16.8‰.

**Table 2:** **Abbreviations and source assignments of bacterial and archaeal intact polar lipids and apolar derivatives**.

**Intact Polar Lipids (core structure)**	**Name**	**Source Assignment**	**References**
**GLYCOLIPIDS**			
1G-, 2G, 2G-P-GDGT (0–4 cyclopentyl rings)	Monoglycosyl-, diglycosyl-, diglycosyl phosphatidyl- glycerol dialkyl glycerol tetraether	Thermophilic archaea	Schouten et al., 2007; Schubotz et al., 2013
G-CER	Glycosyl ceramide	Unknown thermophilic bacteria	Schubotz et al., 2013
1G-DEG, AEG, DAG (C_18_–C_20_)	Monoglycosyl diether, ester/ether, diacylglycerol	Unknown thermophilic bacteria	Bradley et al., 2009; Schubotz et al., 2013
2G-DEG, AEG, DAG (C_18_–C_20_)	Diglycosyl diether, ester/ether, diacylglycerol	Unknown thermophilic bacteria	Bradley et al., 2009; Schubotz et al., 2013
GA-AEG, DAG (C_18_–C_20_)	Glycoronic acid ester/ether, diacylglycerol	Unknown thermophilic bacteria	Bradley et al., 2009; Schubotz et al., 2013
**AMINOGLYCOLIPID**			
G-NG-DAG	Monoglycosyl(N)glycosaminyl diacylglycerol	Unknown thermophilic bacteria	This study
NAcG-2G-DEG	Diglycosyl(N-acetyl)glycosaminyl dietherglycerol	Thermus/Meiothermus	Ferreira et al., 1999
**AMINOPHOSPHOGLYCOLIPID**			
NAcG-P-DEG, DAG	Phospho(N-acetyl)glycosaminyl diether, diacylglycerol	Thermus/Meiothermus, Chtonomonadales	Yang et al., 2006; Lee et al., 2011
**AMINOPHOSPHOLIPID**			
APT-DEG, DAG (C_18_–C_22_)	Aminophosphopentanetetrol diether, diacylglycerol	Aquificae	Sturt et al., 2004
**PHOSPHOLIPIDS**			
PI-DEG, AEG, DAG (C_18_–C_22_)	Phosphatidyl inositol diether, ester/ether, diacylglycerol	Aquificae	Sturt et al., 2004
PE-DAG (C_15_–C_18_)	Phosphatidyl ethanolamine diether, ester/ether, diacylglycerol	Unspecific bacteria	Kates, 1964
PC-DAG C_15_–C_18_)	Phosphatidyl choline diether, ester/ether, diacylglycerol	Unspecific bacteria	Kates, 1964
**AMINOLIPIDS**			
OL, OH-OL (C_15_–C_18_)	Ornithine lipids, hydroxylated ornithine lipids	Unspecific bacteria	Vences-Guzmán et al., 2014
**FATTY ACIDS**			
*i*-, *ai*-C_15_–C_19_		Firmicutes, thermophilic bacteria, Deltaproteobacteria	Kaneda, 1991; van de Vossenberg et al., 1999
C_20:1_, *cy*-C_21:0_, C_20:0_, C_21:0_, C_22:0_		Aquificae	Jahnke et al., 2001

**Figure 2:**
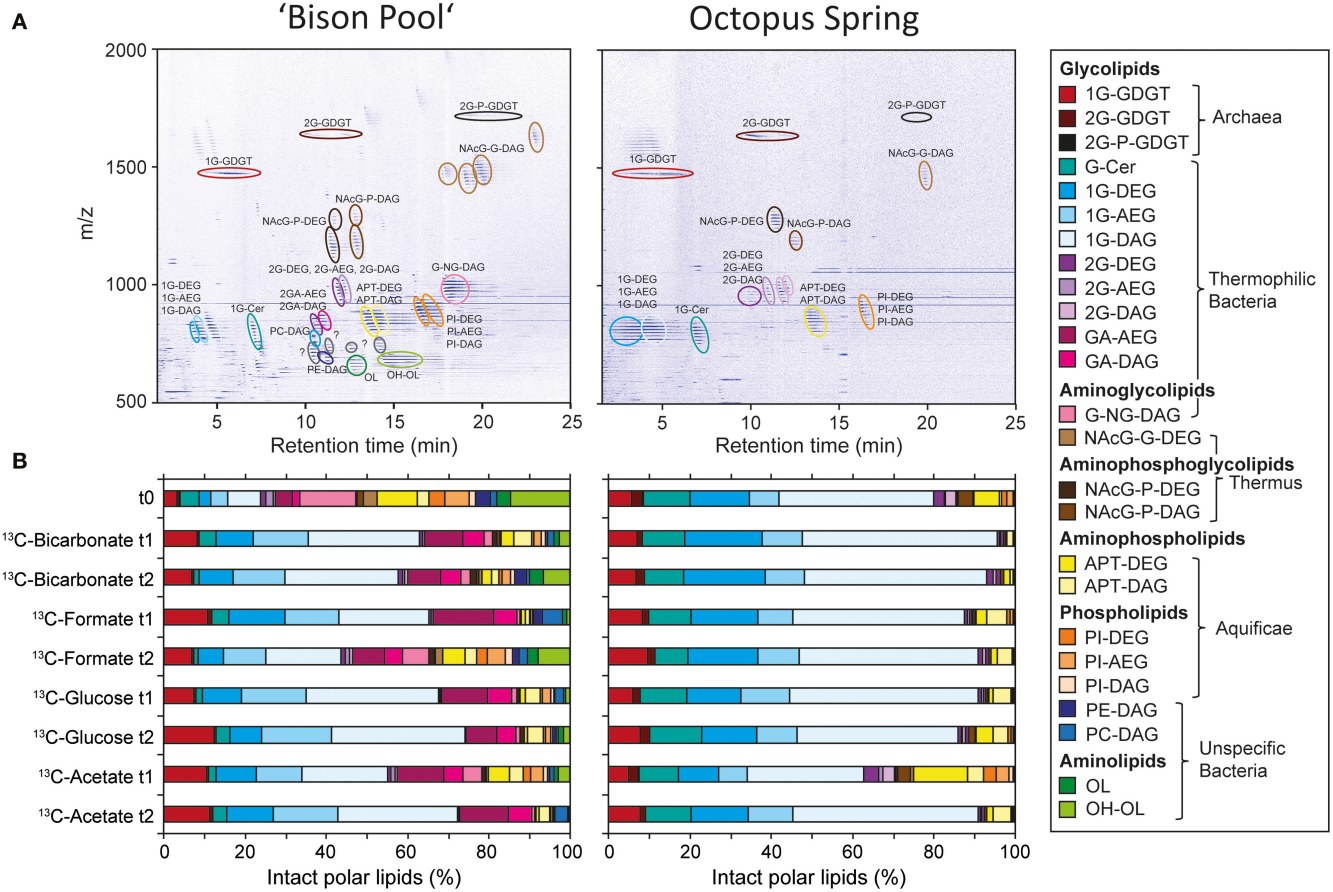
**(A)** Representative HPLC-MS density map chromatograms depicting the intact polar membrane lipid composition of streamer biofilm communities at “Bison Pool” and Octopus Spring at time point zero of the flow-through experiments. **(B)** Changes in intact polar lipid composition during the flow-through experiments at “Bison Pool” and Octopus Spring. For abbreviations of intact polar lipid compound classes and source assignments see Table 2.

Figure 2 shows, representatively from the flow-through experiments, the SBC lipid composition at “Bison Pool” and Octopus Spring (abbreviations and source assignments shown in Table 2). As described previously in Schubotz et al. (2013) both hot spring share similarities in the composition of the major bacterial and archaeal lipid compound groups. Archaea at both springs have predominantly monoglycosidic (1G) and diglycosidic (2G) glycerol dibiphytanyl glycerol tetraether (GDGT) lipids, with minor amounts of GDGTs with phosphatidyl moieties (P-2G-GDGTs). Bacteria at “Bison Pool” and Octopus Spring have diacyl (DAG), diether (DAG) and mixed esther/ether (AEG) glycerol lipids with different headgroups. Glycolipids (1G and 2G), including cerebrosides (Cer), which are glycosylated sphingolipids, are the most abundant intact polar bacterial lipids at both sites, while glycuronic acid (GA) was additionally an abundant compound class at “Bison Pool.” Thermus/Meiothermus-specific diglycosyl(N-acetyl)glycosaminyl (NAcG-2G) and phospho(N-acetyl)glycosaminyl (NAcG-P) as well as Aquificae-diagnostic aminopentanetetrol (APT) and phosphatidylinositol (PI), were found at both sites. At “Bison Pool” also other phospholipids including phosphatidyl choline (PC) and phosphatidyl ethanolamine (PE) and ornithine lipids (OL), sometimes hydroxylated OLs (OH-OL) were detected. Changes in intact polar membrane lipid composition during the course of the flow-through experiments were minimal at Octopus Spring (<10% change in individual lipids). At “Bison Pool” the abundance of individual glycolipids increased by 10–20% compared to the background sample t0, and ornithine lipids decrease by over 10% in some experiments (Figure 2B). Despite these changes in relative abundance, the main lipid compounds of both hot springs were present in all experiments, ensuring that the SBCs composition was not altered significantly during the incubations with different ^13^C-labeled substrates.

**Figure 3:**
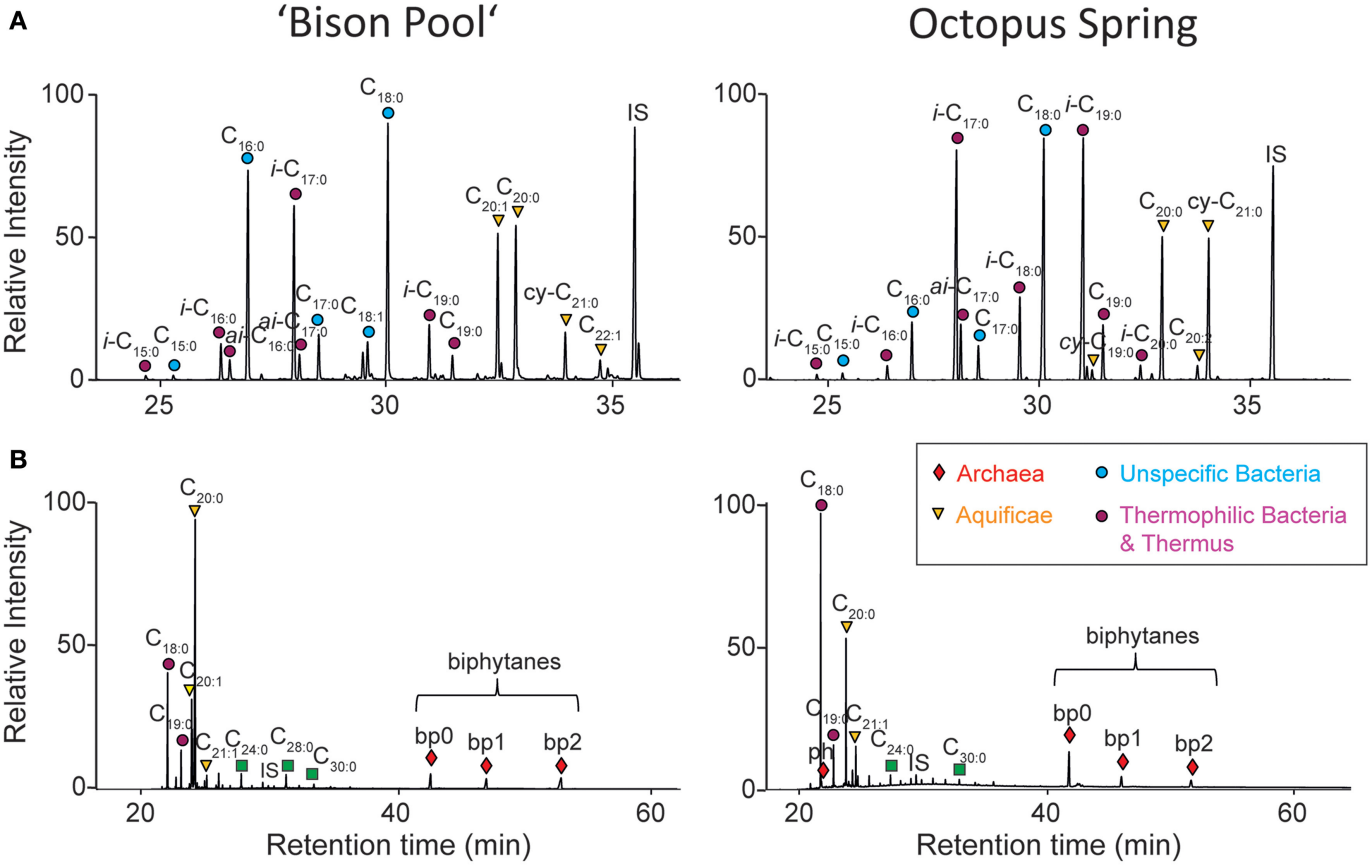
**Representative GC-MS chromatograms of (A) fatty acid methyl esters (FAMEs) and (B) ether cleavage products of streamer biofilm communities at “Bison Pool” and Octopus Spring**. IS, internal standard squalene; ph, phytane; bp, biphytanes.

Representative fatty acid profiles of “Bison Pool” and Octopus Spring (released from the precursor polar lipids by acid hydrolysis) are shown in Figure 3A and have already been described previously (Schubotz et al., 2013). Besides generic C_16:0_ and C_18:0_ fatty acids, both springs have abundant branched fatty acids (e.g., *iso* and *anteiso*-C_17_ and C_19_) and long chain C_20:1_, C_20:0_, and *cy*-C_21:0_ fatty acids. The ether-cleavage products, shown in Figure 3B, are derived from lipids that were originally ether-bound to bacterial diether lipids (C_18:0_–C_21:0_), or archaeal diether (phytane) and tetraether lipids, (biphytanes 0–2).

### Stable carbon isotopic compositions of fatty acids and ether lipids

**Figure 4:**
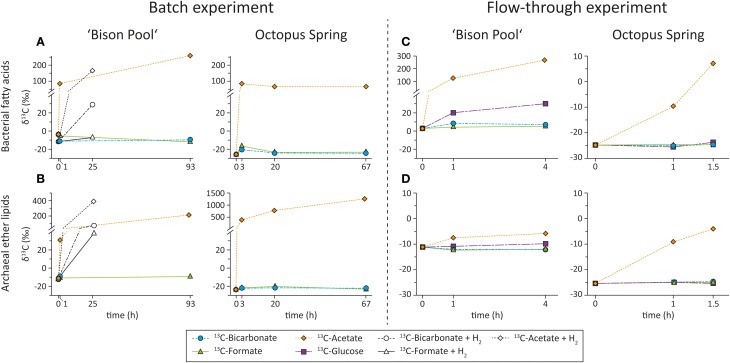
**Development of δ^13^C (expressed as weighted averages) of (A) bacterial fatty acids and (B) archaeal ether lipids in batch-fed experiments at “Bison Pool” and Octopus Spring amended with different ^13^C-labeled substrates**. Incubations at “Bison Pool” were with (open symbols) and without (filled symbols) H_2_ amendments. Development of δ^13^C (expressed as weighted averages) of **(C)** bacterial fatty acids and **(D)** archaeal ether lipids in flow-through experiments at “Bison Pool” and Octopus Spring amended with different ^13^C-labeled substrates.

Figure 4 show the development of the mean isotopic composition of bacterial and archaeal lipids over time in the batch-fed system and flow-through incubations that were carried out over the years 2006, 2007, and 2009. In the batch-fed incubations, intended to identify whether or not we could detect any uptake, SBCs were inoculated with ^13^C-bicarbonate, formate and acetate. In the flow-through reactors, which were intended to more closely mimic the natural settings, incubations were continuously fed with the ^13^C-labeled bicarbonate, formate and glucose.

#### Batch-fed incubations without amendment of electron donor

The incubations with ^13^C-bicarbonate, formate and acetate that were carried out in an aqueous environment, open to the atmosphere at both “Bison Pool” and Octopus Spring showed highest label uptake with acetate (Table 3, Figure 4). After 1 h at “Bison Pool” there was an increase of over 90‰ in the weighted mean isotopic composition (δ^13^C_wm_) of the major bacterial fatty acids, which increased to over 200‰ after 93 h (Figure 4). For archaeal ether lipids Δ δ^13^C_wm_ was over 40‰ at “Bison Pool” after 1 h and over 200‰ after 93 h. No significant isotopic change was observed with bicarbonate or formate for individual bacterial FAMEs. However, a small isotopic change in archaeal lipids was detected, specifically in phytane (which is the ether cleavage product of archaeol), with a significant isotopic enrichment of over 10 and 6‰ for ^13^C-bicarbonate and formate, respectively (Table 3). Bacterial diether lipids exhibited up to 10‰ increases of Δ δ^13^C_wm_ at “Bison Pool” with ^13^C-acetate, but not for any of the other substrates (Table 3). At Octopus Spring, isotopic enrichments with acetate was even higher than at “Bison Pool” with Δ δ^13^C_wm_ of over 110 and 400‰ in bacterial FAMEs and archaeal ether lipids, respectively, after only 3 h. Increase in Δ δ^13^C_wm_ of archaeal lipids continued after 3 days, with a final isotopic enrichment over 1280‰. For bacterial lipids a decrease in label uptake was observed after 3 h with final Δ δ^13^*C*_wm_-values of 94‰. No significant isotopic changes were observed for bacterial diether lipids for any of the ^13^C-labeled substrates at Octopus Spring (Table 3).

**Table 3:** **Stable carbon isotopic compositions of bacterial and archaeal lipids in batch-fed experiments inoculated with ^13^C-bicarbonate, formate and acetate**.

	**“Bison Pool”**								**Octopus Spring**							
			**^**13**^C-Bicarb**.		**^**13**^C-Formate**		**^**13**^C-Acetate**				**^**13**^C-Bicarb**.		**^**13**^C-Formate**		**^**13**^C-Acetate**	
	t0^*^		**93 h**		93 h^**^		**93 h**		**t0**		**67 h**		**67 h**		**67 h**	
	**%**	**δ^**13**^C(‰)**	**%**	**δ^**13**^C(‰)**	**%**	**δ^**13**^C(‰)**	**%**	**δ^**13**^C(‰)**	**%**	**δ^**13**^C(‰)**	**%**	**δ^**13**^C(‰)**	**%**	**δ^**13**^C(‰)**	**%**	**δ^**13**^C(‰)**
**FATTY ACIDS**																
*i*-C_15:0_	<0.1	−	3.8	−6.8	1.5	−12.7	1.2	893	0.3	−25.1	−	−	−	−	−	-
*ai*-C_15:0_	<0.1	−	0.5	−	0.7	−25.5	<0.1	−	−	−	−	−	−	−	−	-
C_15:0_	<0.1	−	0.2	−	0.5	−29.1	<0.1	−	1.0	−18.7	−	−	−	−	−	-
*i*-C_16:0_	0.2	−15.7	6.7	−8.2	1.8	−13.3	1.8	1038	0.8	−32.1	0.4	−24.9	0.1	−23.9	−	-
C_16:1_	<0.1	−	1.0	−21.8	1.1	−27.0	0.2	−	−	−	−	−	0.6	−	0.8	367
C_16:0_	3.2	−24.9	9.1	−25.2	9.2	−27.6	6.3	92.6	13.2	−28.3	2.4	−27.9	2.0	−27.7	2.4	60.6
*i*-C_17:0_	7.9	−9.4	22.7	−7.1	11.3	−9.4	11.1	936	15.2	−26.4	19.8	−24.6	22.1	−23.7	26.5	150
*ai*-C_17:0_	0.4	−	3.8	−11.8	1.9	−18.0	1.3	496	2.6	−24.1	2.7	−22.1	2.2	−20.3	2.8	276
C_17:0_	0.7	−9.9	0.6	−	0.7	−21.8	0.6	−	3.3	−19.4	1.2	−24.0	1.3	−25.0	1.0	85.0
*i*-C_18:0_	1.1	−14.1	0.4	−12.2	1.0	−20.5	1.0	452	4.1	−26.7	6.4	−25.4	5.4	−25.3	6.0	53.9
C_18:1_	2.1	−9.0	9.8	−24.1	13.6	−27.0	2.7	56.9	2.3	−25.3	2.5	−25.3	−	−	−	-
C_18:0_	20.2	−3.4	10.7	−10.8	11.9	−11.4	17.7	170	11.7	−22.8	16.0	−24.5	14.6	−24.1	13.1	63.0
*i*-C_19:0_	8.8	−7.9	4.9	−3.6	5.5	−8.3	5.1	916	14.5	−26.8	24.5	−24.9	25.4	−24.7	24.8	17.0
*ai*-C_19:0_	1.0	−	0.6	−	0.6	−7.0	0.4	−	0.7	−24.0	1.4	−22.1	1.1	−21.4	1.2	108
*cy*-C_19:0_	0.3	−	0.2	−	0.2	−	0.2	−	0.9	−23.1	0.3	−	0.5	−	0.4	15.6
C_19:0_	2.2	−11.0	0.8	−	0.9	−5.5	0.9	251	1.5	−23.4	2.4	−23.7	2.2	−23.3	1.9	15.9
*i*-C_20:0_	−	−	−	−	−	−	−	−	2.7	−21.4	0.9	−22.3	0.6	−20.2	0.7	30.2
C_20:1_	34.5.	2.9	15.8	0.8	23.9	1.9	30.4	40.1	0.8	−24.3	2.2	−24.2	0.8	−17.2	0.8	-16.3
C_20:0_	10.2	−2.6	3.5	−5.5	5.6	−5.4	6.6	137	2.7	−23.7	8.3	−25.5	8.1	−24.5	6.1	5.3
C_20:2_	−	−	−	−	−	−	−	−	0.7	−26.2	0.6	−	0.6	−	0.6	-
*cy*-C_21:0_	3.8	2.2	2.5	−0.4	3.6	−1.5	6.3	107	5.1	−23.5	4.8	−23.3	11.0	−23.6	9.6	-13.9
C_22:1_	1.6	−15.0	1.8	−24.1	3.5	−25.7	5.6	−21.0	−	−	1.7	−28.3	0.4	−	0.4	-
C_22:0_	1.5	−29.2	0.6	−	1.0	−30.3	0.6	−	−	−	1.5	−29.1	1.0	−	0.9	-
Weighted mean		−3.5		−9.6		−12.0		257		−25.1		−24.6		−23.3		69.0
**BACTERIAL DIETHERS**																
C_18:0_/C_18:0_ DEG	19.9	−1.3	20.7	−2.5	14.8	−3.1	16.0	7.3	29.6	−24.9	43.9	−22.6	25.5	−23.3	30.0	-21.3
C_18:0_/C_20:1_ DEG	43.4	1.1	70.3	−1.7	63.1	−1.4	67.6	10.8	16.1	−23.0	20.3	−22.7	14.5	−23.3	9.5	-23.5
C_20:0_/C_20:1_ DEG	32.8	−1.7	5.8	−4.2	21.0	−4.6	14.8	16.5	44.7	−25.1	22.4	−22.9	50.4	−23.9	54.2	-22.9
Weighted mean		0.8		−2.4		−2.3		11.1		−23.7		−22.2		−19.9		-20.1
**ARCHAEAL DIETHERS**																
Archaeol		−14.0		*nd*		*nd*		*nd*		−26.7		−19.4		−16.0		1292
**ETHER CLEAVAGE PRODUCTS**																
Phytane	11.1	−14.1	11.2	−3.0	6.7	−8.5	5.1	1447	3.8	−25.0	4.4	−20.4	4.2	−18.3	3.3	1210
Biphytane 0	48.3	−9.8	58.4	−8.3	34.1	−6.7	51.1	263	55.7	−25.5	64.0	−23.4	63.8	−24.5	67.1	1679
Biphytane 1	14.7	−9.8	16.1	−11.0	24.9	−9.1	21.4	46	23.4	−21.7	19.0	−21.1	19.6	−22.0	18.8	490
Biphytane 2	25.9	−13.1	14.3	−14.7	34.3	−12.6	22.4	−10.2	17.1	−19.8	12.6	−19.0	12.4	−19.0	10.8	18.3
Weighted mean		−11.1		−9.0		−9.5		215.8		−23.7		−22.2		−23.0		1263.9

nd, no data,

* t0 at “Bison Pool” is a SBC sample collected in 2005 (Schubotz et al., 2013).

** For the ether cleavage products there is no data for ^*13*^C-bicarbonate time point 93 h, therefore time point isotopic compositions after 1 h are reported.

#### Batch-fed incubations with amendment of H_2_

Amendment of H_2_ as energy source resulted in significant label uptake with both ^13^C-acetate and ^13^C-bicarbonate into bacterial FAMEs after 1 day (Figure 4, Table 4), with Δ δ^13^C_wm_ 170 and 40‰ for the bacterial fatty acids, respectively. Formate showed only a slight increase in δ^13^C_wm_ of FAMEs with H_2_ amendment of 4‰. For bacterial diether lipids no significant changes in isotopic composition were observed with ^13^C-bicarbonate and ^13^C-formate, with ^13^C-acetate there were minor increases in Δ δ^13^C_wm_ of 2.8‰ (Table 4). Conversely, archaeal ether lipids exhibited very large isotopic changes with both ^13^C-bicarbonate (88‰) and ^13^C-formate (50‰) after H_2_ addition. Unfortunately there is no data for the ether-cleaved ^13^C-acetate sample; however, an isotopic change of archaeol of over 400‰ indicates that also acetate uptake into archaeal lipids was immensely stimulated after addition of H_2_ (Table 4).

**Table 4:** **Stable carbon isotopic compositions of bacterial and archaeal lipids in batch-fed experiments inoculated with ^13^C-bicarbonate, formate and acetate**.

	**“Bison Pool”**							
			**^**13**^C-Bicarb**.		**^**13**^C-Formate**		**^**13**^C-Acetate**	
	**t0**		**25 h**		**25 h**		**25 h**	
	**%**	**δ^**13**^C (‰)**	**%**	**δ^**13**^C (‰)**	**%**	**δ^**13**^C (‰)**	**%**	**δ^**13**^C (‰)**
**FATTY ACIDS**								
*i*-C_15:0_	3.5	−7.3	4.0	84.0	5.8	−5.6	4.1	308
*ai*-C_15:0_	0.5	−14.8	0.6	53.0	1.0	−13.0	0.6	365
C_15:0_	0.2	−	0.2	−	−	−	0.3	−
*i*-C_16:0_	5.9	−8.9	7.5	55.3	12.4	−7.4	8.3	284
C_16:1_	2.7	−20.0	1.3	3.7	0.1	−7.1	1.4	76.4
C_16:0_	11.2	−25.3	4.9	−3.2	7.1	−19.8	6.8	79.3
*i*-C_17:0_	16.9	−7.6	22.1	76.6	22.4	−6.6	19.2	344
*ai*-C_17:0_	3.2	−11.7	3.7	31.4	5.7	−11.4	4.0	349
C_17:0_	0.5	−12.2	0.6	52.9	0.5	−	0.6	286
*i*-C_18:0_	9.5	−23.6	0.3	32.5	2.4	−10.2	4.8	79.7
C_18:1_	9.5	−26.5	5.2	−8.9	4.4	−15.6	7.3	-8.8
C_18:0_	9.3	−8.9	10.5	4.8	10.8	−5.6	10.0	132
*i*-C_19:0_	2.6	−8.3	5.2	39.9	2.6	−7.4	3.7	323
*ai*-C_19:0_	0.4	−10.4	0.5	22.4	0.4	−9.0	0.6	323
*cy*-C_19:0_	0.2	−10.8	0.2	−	−	−	0.2	216
C_19:0_	0.5	−4.9	0.9	14.5	0.6	−	0.8	130
−C_20:0_	−		−	−	−	−	−	−
C_20:1_	17.0	2.5	21.2	3.8	14.3	1.6	16.8	12.1
C_20:0_	3.1	−3.7	4.6	0.4	3.1	−3.7	2.3	56.4
C_20:2_	−	−	−	−	−	−	−	−
*cy*-C_21:0_	3.3	0.2	4.7	2.2	3.7	0.1	3.7	6.4
C_22:1_	<0.1	−12.5	1.5	−23.4	2.7	−20.6	4.0	-0.7
C_22:0_	<0.1	−26.6	0.3	−27.3	<0.1	−25.6	0.5	-9.1
Weighted mean		−11.5		29.1		−7.1		161
**BACTERIAL DIETHERS**								
C_18:0_/C_18:0_ DEG	20.7	−1.3	21.1	0.2	22.7	−0.6	22.3	1.0
C_18:0_/C_20:1_ DEG	45.2	1.1	47.4	1.2	48.6	1.1	37.5	2.0
C_20:0_/C_20:1_ DEG	34.1	−1.7	31.5	0.4	28.7	−1.3	40.2	3.8
Weighted mean		−0.3		0.7		0.0		2.5
**ARCHEAL DIETHERS**								
Archaeol	4.0	−14.0	4.9	252	8.7	−13.3	5.3	396
**ETHER CLEAVAGE PRODUCTS**								
Phytane	11.1	−14.1	11.1	249.9	18.8	195.1	18.4	*nd*
Biphytane 0	48.3	−9.8	48.3	109.6	18.2	42.7	30.8	*nd*
Biphytane 1	14.7	−9.8	14.7	−1.0	29.2	−5.6	20.6	*nd*
Biphytane 2	25.9	−13.1	25.9	−13.6	33.8	−12.9	30.2	*nd*
Weighted mean		−11.1		77.0		38.4		−

Experiments at “Bison Pool” were amended with H_2_. nd, no data.

#### Flow through reactor incubations

In addition to ^13^C-bicarbonate, formate and acetate SBCs were also incubated with ^13^C-glucose in the flow-through experiments (Figure 4, Table 5). Comparable to the batch-fed incubations, the largest isotopic changes for bacterial FAMEs occurred with acetate as substrate resulting in Δ δ^13^C_wm_ of 32‰ after 1.5 h at Octopus Spring and 275‰ after 4 h at “Bison Pool.” Incubations with ^13^C-bicarbonate and formate resulted in insignificant isotopic enrichments for bacterial FAMEs at “Bison Pool,” 2.9 and 1.9‰, respectively, while a significant increase in Δ δ^13^C_wm_ of FAMEs was observed with glucose (19‰). At Octopus Spring there were no significant isotopic changes in bacterial FAMEs for any of the other substrates (Table 5). In comparison with the batch-fed incubations, archaeal ether lipids at “Bison Pool” and Octopus Spring showed little ^13^C-incorporation with acetate or the other labeled substrates. Significant isotopic enrichments for archaeal lipids were only found in the ^13^C-acetate experiment, particularly, in archaeol/phytane (139/250‰) and biphytane 0 (17‰) at Octopus Spring and phytane (25‰) at “Bison Pool.”

**Table 5:** **Stable carbon isotopic compositions of bacterial and archaeal lipids in flow-through reactors inoculated with ^13^C-bicarbonate, formate, acetate and glucose**.

	**“Bison Pool”**										**Octopus Spring**									
			**^**13**^C-Bicarb**.		**^**13**^C-Formate**		**^**13**^C-Acetate**		**^**13**^C-Glucose**				**^**13**^C-Bicarb**.		**^**13**^C-Formate**		**^**13**^C-Acetate**		**^**13**^C-Glucose**	
	**t0**		**4 h**		**4 h**		**4 h**		**4 h**		**t0**		**1.5 h**		**1.5 h**		**1.5 h**		**1.5 h**	
	**ng g^−**1**^**	**δ^**13**^C (‰)**	**ng g^−**1**^**	**δ^**13**^C (‰)**	**ng g^−**1**^**	**δ^**13**^C (‰)**	**ng g^−**1**^**	**δ^**13**^C (‰)**	**ng g^−**1**^**	**δ^**13**^C (‰)**	**ng g^−**1**^**	**δ^**13**^C (‰)**	**ng g^−**1**^**	**δ^**13**^C (‰)**	**ng g^−**1**^**	**δ^**13**^C (‰)**	**ng g^−**1**^**	**δ^**13**^C (‰)**	**ng g^−**1**^**	**δ^**13**^C (‰)**
**FATTY ACIDS**																				
i-C_15:0_	40.1	−6.6	992	−4.3	542	−6.2	1141	366	1213	20.0	18.1	−26.9	14.0	−26.8	26.0	−25.5	7.8	178	14.9	−20.4
ai-C_15:0_	5.9	−16.8	166	−11.7	43.2	−8.2	70.3	2283	85.4	188.7	−	−	−	−	−	−	−	−	−	−
C_15:0_	40.8	−17.4	108	−13.8	278	−16.6	59.0	1161	186	14.1	23.5	−20.6	4.9	−21.8	18.5	−22.0	7.7	−2.2	15.0	−20.8
i-C_16:0_	252	−9.2	1837	−7.6	977	−7.5	1630	389	2080	25.1	45.7	−24.6	34.0	−25.3	56.1	−24.5	21.9	150	51.6	−16.8
C_16:1_	144	−26.6	484	−21.1	43.2	−12.3	136	−10.0	202	−17.2	−	−	−	−	−	−	−	−	−	−
C_16:0_	1585	−25.1	2854	−20.9	2126	−18.1	1328	929	1523	43.2	182	−25.6	169	−26.7	215	−26.1	257	−18.1	195	−25.3
i-C_17:0_	178	−7.7	6044	−6.3	3719	−6.9	5789	621	6608	22.4	877	−26.5	930	−26.4	1439	−25.8	570	26.4	1464	−24.8
ai-C_17:0_	69.0	−11.0	711	−10.1	402	−7.2	581	1687	845	95.4	158	−22.7	81.3	−23.5	117	−21.8	74.9	89.0	151	−14.5
C_17:0_	319	−16.6	333	−9.6	1253	−15.7	300	724	534	9.4	101	−22.0	51.5	−22.7	92	−22.3	42.3	15.1	91.8	−21.1
i-C_18:0_	187	−10.5	709	−8.0	397	−7.3	555	905	736	38.1	255	−25.7	194	−26.6	313	−26.4	128	24.1	325	−24.5
C_18:1_	260	−23.8	1034	−14.4	350	−13.9	689	61.1	510	−2.4	−	−	−	−	−	−	−	−	−	−
C_18:0_	2019	−6.4	6865	−1.2	4732	−1.1	7953	218	8182	7.5	930	−23.5	558	−23.8	911	−23.3	426	5.9	-36	−22.8
i-C_19:0_	368	−7.3	1621	−7.2	955	−6.5	1437	624	1611	0.5	969	−26.5	685	−26.1	1122	−26.1	483	−2.4	1108	−25.7
ai-C_19:0_	35.7	−3.5	218	0.5	76.4	−1.8	272	609	221	26.7	37.5	−25.3	27.9	−25.3	39.3	−22.9	20.6	44.7	43.7	−22.5
cy-C_19:0_	31.2	−16.4	61.6	−8.4	43.2	−9.9	69	63.6	55.4	−4.6	32.5	−22.9	52.1	−25.3	95.5	−25.5	15.3	−15.2	49.8	−23.8
C_19:0_	174	−3.2	540	−1.1	397	−2.5	641	106	703	1.4	172	−22.6	96.8	−22.8	162	−22.6	68.8	−0.5	168	−22.1
iC_20:0_	−	−	−	−	−	−	−	−	−	−	43.9	−27.1	25.0	−26.7	43.0	−23.4	18.6	8.7	39.9	−24.7
C_20:1_	1054	−4.4	8901	4.7	2022	5.1	12966	28.7	8856	5.1	20.7	−23.2	32.7	−29.7	63.7	−23.7	34.5	23.0	23.4	−21.6
C_20:0_	1252	−2.1	3524	1.6	2628	1.1	4223	68.3	4451	3.9	493	−23.5	318	−23.7	443	−25.0	266	−17.7	448	−23.2
C_20:2_	40.1	0.5	90.4	2.6	43.2	2.1	136	32.6	123	3.6	45.0	−25.5	23.6	−25.9	39.0	−25.0	20.6	0.8	40.9	−25.0
cy-C_21:0_	339	0.8	1449	1.0	1332	1.0	1941	7.8	2007	1.3	485	−23.4	756	−23.9	1387	−23.3	212	−20.9	638	−22.8
C_22:1_	5.9	2.2	539	3.2	180	3.1	340	24.9	659	3.3	−	−	−	−	−	−	−	−	−	−
C_22:0_	295	−22.9	1315	−9.2	184	−15.6	816	18.8	1674	−8.9	−	−	−	−	−	−	−	−	−	−
Weighted mean		−6.5		−3.6		−4.8		268		12.8		−24.8		−24.8		−24.8		7.1		−23.8
**ETHER CLEAVAGE PRODUCTS**																				
C_18:0_	609	1.7	131	1.1	814	−1.0	28.1	11.0	43.5	2.7	1385	−23.2	1207	−23.6	1269	−23.3	429	−20.1	1628	−25.0
C_19:0_	167	−1.5	133	−1.5	100	−6.8	33.8	129.6	14.6	3.4	385	−23.8	254	−22.1	310	−24.0	300	−22.0	394	−24.7
C_20:1_	720	3.7	997	5.1	852	2.7	398	103.9	311.6	9.3	−	−	88.6	−	91.2	−	38.0	−	116	−
C_20:0_	2202	2.1	1955	2.0	506	1.3	909	5.7	873	3.3	1318	−23.3	1946	−22.6	1276	−23.1	699	−23.4	1481	−23.0
C_21:1_	47.4	−2.3	146	−2.5	−	−	66.7	14.0	57.0	−	565	−24.8	570	−24.8	495	−24.6	314	−27.0	632	−26.4
Phytane	195	−14.0	35.2	−8.4	45.5	−10.9	11.9	20.9	24.1	−6.3	78.7	−25.0	200	−25.7	73.8	−26.7	25.6	223.0	120	−24.2
Biphytane 0	203	−8.6	542	−8.9	905	−9.8	483	−10.1	476	−10.1	1208	−27.2	1689	−27.6	990	−27.1	736	−10.3	1740	−27.0
Biphytane 1	168	−10.1	902	−11.7	1298	−10.8	777	−11.7	907	−10.7	537	−24.1	610	−24.2	354	−22.9	264	−22.9	627	−23.5
Biphytane 2	266	−13.0	1753	−13.6	2447	−13.4	1524	−14.1	1174	−13.4	468	−20.2	461	−22.8	286	−19.8	219	−19.1	541	−19.4
Weighted mean		−1.2		−5.7		−8.2		−0.9		−0.4		−25.3		−24.5		−25.0		−4.1		−25.4

nd, no data.

### Uptake of organic and inorganic carbon into bacterial and archaeal biomass

**Figure 5:**
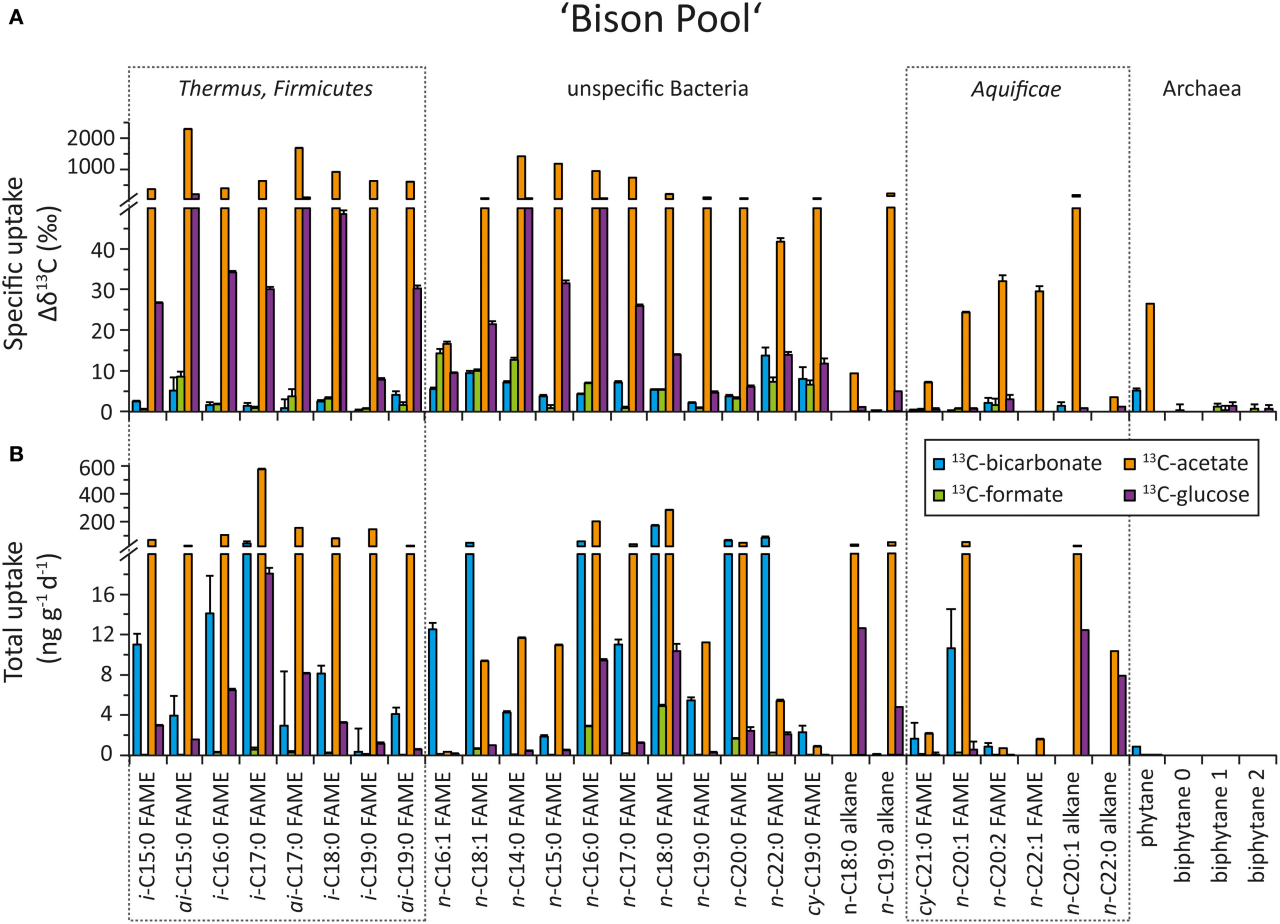
**Uptake of ^13^C-labeled substrates into different bacterial and archaeal lipids during the flow-through experiment at “Bison Pool.”** For source assignments of diagnostic lipids see Table 2. **(A)** Carbon isotopic change of individual fatty acids and selected archaeal ether lipids after incubation with ^13^C-labeled bicarbonate (blue), formate (green), acetate (orange), and glucose (purple) at the end of the experiment. The errors were determined as the sum of the analytical standard deviations (determined by triplicate measurements). **(B)** Carbon assimilation into bacterial and archaeal lipids based on carbon isotopic changes at the end of the experiment. Errors were determined through Gaussian error propagation of the analytical standard deviation (for details see text).

The top panel of Figures 5 (“Bison Pool”) and 6 (Octopus Spring) show the specific uptake, which is defined as the relative change in carbon isotopic compositions (Δ δ^13^C) of major bacterial and archaeal lipids, from the start to the end of the flow-through experiment. Both at “Bison Pool” and Octopus Spring, the strongest label incorporation again resulted from the ^13^C-acetate amendments, followed by the ^13^C-glucose experiments. However, the incorporation of label was different for each lipid. For fatty acids, highest enrichments were found in branched FAMEs, particularly *ai*-C_15:0_ and *ai*-C_17:0_ at “Bison Pool” and *i*-C_15:0_, *i*-C_16:0_ and *ai*-C_17:0_ at Octopus Spring. At “Bison Pool” saturated C_14:0_, C_15:0_, C_16:0_, and C_17:0_ fatty acids were also highly labeled. Label incorporation overall was lowest in the ^13^C-bicarbonate and formate experiments: at “Bison Pool” Δ δ^13^*C*-values were over 100 times lower than with acetate and ^13^C-uptake was mainly found for C_16:1_ and C_18:1_ fatty acids. At Octopus Spring, the ^13^C-bicarbonate and formate experiments resulted in minor labeling of branched fatty acids. Among archaeal lipids, archaeol and its derivative phytane showed the highest label incorporation at both hot springs with ^13^C-acetate. At Octopus Spring, biphytane 0 was also significantly labeled in the acetate experiment, while the biphytanes at “Bison Pool” did not show any label uptake in any experiment. However, minor ^13^C-bicarbonate uptake was detected in phytane at both Octopus Spring and “Bison Pool.”

**Figure 6:**
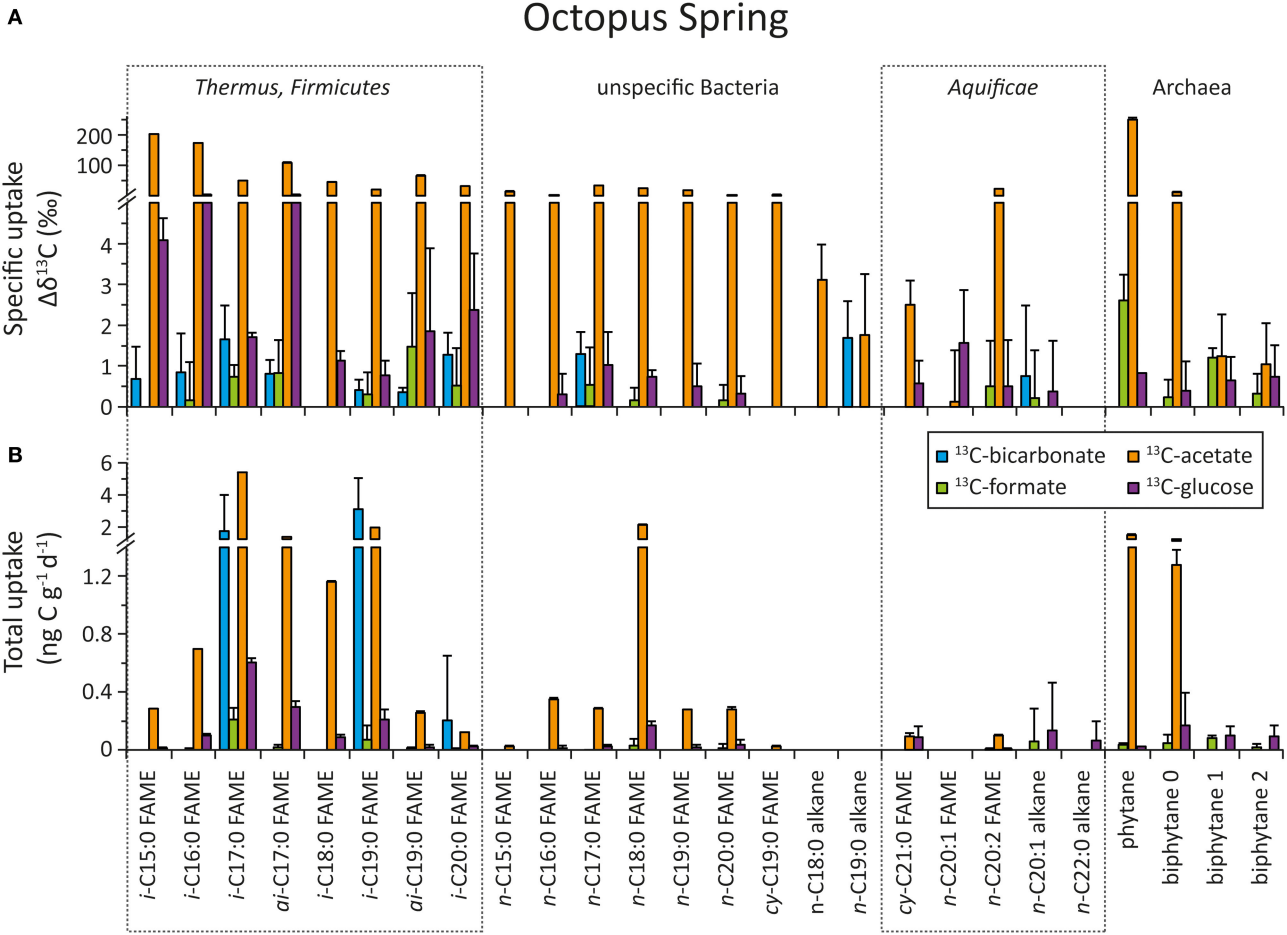
**Uptake of ^13^C-labeled substrates into different bacterial and archaeal lipids during the flow-through experiment at Octopus Spring**. For source assignments of diagnostic lipids see Table 2. **(A)** Carbon isotopic change of individual fatty acids and selected archaeal ether lipids after incubation with ^13^C-labeled bicarbonate (blue), formate (green), acetate (orange), and glucose (purple) at the end of the experiment. The errors were determined as the sum of the analytical standard deviations (determined by triplicate measurements). **(B)** Carbon assimilation into bacterial and archaeal lipids based on carbon isotopic changes at the end of the experiment. Errors were determined through Gaussian error propagation of the analytical standard deviation (for details see text).

Total carbon uptake rates (ng C g^−1^ day^−1^), determined by taking into consideration absolute concentrations of individual lipids (Table 5) and % ^13^C-label of the substrates in each experiment, are summarized in Table 6. The bottom panels of Figures 5, 6 show the carbon uptake rate into individual lipids. At “Bison Pool” total carbon uptake into bacterial and archaeal lipids was highest for acetate with 1848 ± 6.1 ng g^−1^ day^−1^, followed by bicarbonate with 555.9 ± 64.3 ng g^−1^ day^−1^ and glucose 71.0 ± 3.9 ng g^−1^ day^−1^, uptake with formate was very low (13.1 ± 1.0 ng g^−1^ day^−1^). At Octopus Spring total carbon uptake into bacterial and archaeal lipids was also highest for acetate at 17.0 ± 0.4 ng g^−1^ day^−1^, followed by bicarbonate, albeit with a large error of uncertainty (5.3 ± 4.6 ng g^−1^ day^−1^) and glucose (2.0 ± 0.7 ng g^−1^ day^−1^), uptake with formate was within the range of the analytical error 0.6 ± 0.4 ng g^−1^ day^−1^.

**Table 6:** **Total carbon uptake into bacterial, archaeal and total lipids at Bison Pool and Octopus Spring with ^13^C-bicarbonate, formate, acetate and glucose**.

	**“Bison Pool”**				**Octopus Spring**			
	**Bicarbonate**	**Formate**	**Acetate**	**Glucose**	**Bicarb**.	**Formate**	**Acetate**	**Glucose**
**ng C g^−**1**^ day^−**1**^**								
Bacterial lipids	554 ± 64.2	13.1 ± 1.0	1848 ± 6.1	71.0 ± 3.9	5.3 ± 4.6	0.4 ± 0.3	14.1 ± 0.3	2.0 ± 0.7
Archaeal lipids	0.9 ± 0.1	0.02 ± 0.01	0.07 ± 0.01	0.02 ± 0.01	−	0.2 ± 0.1	2.9 ± 0.2	0.02 ± 0.01
Total lipids	555 ± 64.3	13.1 ± 1.0	1848 ± 6.1	71.0 ± 3.9	5.3 ± 4.6	0.6 ± 0.4	17.0 ± 0.4	2.0 ± 0.7

In the ^13^C-acetate experiments the greatest carbon uptake was observed for branched *i*- and *ai*-fatty acids and generic fatty acids C_16:0_ and C_18:0_ at “Bison Pool” (>20 ng g^−1^ day^−1^). At Octopus Spring acetate uptake was mainly observed into branched fatty acids and *n*-C_18:0_. Octopus Spring also showed comparably elevated carbon assimilation into archaeal ether lipids (phytane and biphytane 0) with acetate as substrate (>1 ng g^−1^ day^−1^). Although only small isotopic changes were observed for the ^13^C-bicarbonate experiment, the low % ^13^C-label of the “Bison Pool” experiments results in large carbon uptake rates for even-carbon-numbered saturated fatty acids (C_16_–C_22_), C_18:1_ and *i*-C_17:0_(>20 ng g^−1^ day^−1^). Bicarbonate carbon assimilation was also observed for other branched and monounsaturated fatty acids (Figure 5), but at a lower rate. At Octopus Spring carbon uptake from ^13^C-bicarbonate was less than at “Bison Pool” and occurred mainly into *i*-C_17:0_*i*-C_19:0_ and *i*-C_20:0_ fatty acids. Notably, the error calculations indicate a large uncertainty in these values. Carbon uptake into individual lipids with ^13^C-formate was minor at “Bison Pool” where it occurred mainly into C_16:0_, C_18:0_, and C_20:0_ fatty acids (>1.5 ng g^−1^ day^−1^), and was within the range of the analytical error at Octopus Spring.

## Discussion

There is a very long history behind our interest in the metabolism of SBCs dating back to Setchell (1903) who recognized “chlorophylless Schizomycetes” as bacteria living in siliceous hot springs at temperatures up to 89°C (Setchell, 1903). Subsequent investigations demonstrated that filamentous bacteria colonized glass slides immersed in the outflow channel of Octopus Spring at temperatures up to 91°C (Brock, 1978). The first cultured isolate from this community, *Thermocrinis ruber*, was shown to exhibit considerable metabolic diversity and grew chemolithoautotrophically with multiple electron donors, including hydrogen, thiosulfate, and elemental sulfur, and with oxygen as the electron acceptor. Furthermore, formate and formamide could provide energy and carbon sources under aerobic conditions (Huber et al., 1998; #2462). In subsequent investigations, however, C-isotopic analyses of *Thermocrinis*-specific lipids in SBC from Octopus Spring, did not provide an unequivocal answer to the question about whether natural “streamers” engaged in autotrophic growth or heterotrophic growth (Jahnke et al., 2001). For the first time, the current study provides direct evidence from carbon isotopic labeling experiments that natural SBC primarily grow heterotrophically, but with the potential for mixotrophic growth. During heterotrophic growth, formate does not seem to be the main source of carbon in this system. Instead acetate and glucose were metabolized more readily, accounting for over 75% of total carbon uptake during the flow-through experiments (Table 6). Notable differences in carbon uptake into diagnostic lipids were observed between the two alkaline hot springs and are discussed further below.

### Microbial lipid patterns and their stable carbon isotopic compositions in streamer biofilm communities

SBCs are known to contain unique lipid biosignatures that can be traced back to distinct thermophilic source organisms (Table 2). All of the previously described signature lipids could be detected in the SBC with some differences in relative abundance between “Bison Pool” and Octopus Spring. For instance, in the 2009 flow-through control sample, Aquificae-diagnostic head groups APT and PI and were more abundant at “Bison Pool” (ca. 15%) than Octopus Spring (ca. 10%), while Thermus/Meiothermus diagnostic head groups NAcG-2G and NAcG-P were equally abundant at both sites (ca. 5%). Both, Octopus Spring and “Bison Pool” were dominated by glycolipids with mixed ester and ether core structures of a yet unassigned thermophilic bacterial origin (Table 2). Differences in the bacterial head group composition are also reflected in the fatty acid profiles for the years 2006, 2007, and 2009, with C_20:1_ = C_20:0_ > *cy*-C_21:0_ being the predominant Aquificae-diagnostic fatty acids at “Bison Pool” and C_20:0_ = *cy*-C_21:0_ > C_20:2_ > cyC_19:0_ at Octopus Spring (Figure 3, Tables 3, 5). Branched *iso* and *anteiso* fatty acids, which are known to be synthesized by Thermus/Meiothermus, Firmicutes, or Deltaproteobacteria (Taylor and Parkes, 1985; Kaneda, 1991; Yang et al., 2006) were relatively more abundant at Octopus Spring than Bison Pool, with *i*-C_19:0_ and *i*-C_17:0_ being typically highest (Figure 3, Tables 2, 5). The observed differences in fatty acid compositions between the two hot springs could be due to changes in temperatures or due to shifts within the SBC community, which in turn might synthesize branched or long chain fatty acids in different abundances.

In addition to inferences about source-organisms the signature lipids contain specific isotopic information that yield clues concerning carbon assimilation pathways of these organisms. The SBC samples analyzed in this study confirms previous observations that the same fatty acid or ether lipid biomarkers contain contrasting compound-specific stable carbon isotopic compositions at “Bison Pool” and Octopus Spring (cf., Schubotz et al., 2013), with SBC lipids at Octopus Spring being comparably lighter than an “Bison Pool” (Tables 3–5). Taking into account the similar δ^13^*C*-values (and concentration) of the DIC and DOC carbon pools at both springs (Table 1), with δ^13^C of DOC at Ocotopus Spring only being slightly lighter than “Bison Pool” (2–7‰), implies that the SBCs at each spring must have inherently distinct carbon sources and/or are able to switch the modes of carbon metabolism depending on carbon inputs.

### Carbon uptake into streamer biofilm communities

All experiments showed highest ^13^C-label uptake with acetate into both bacterial and archaeal lipids, indicating that heterotrophy is a major carbon assimilation pathway of the SBCs at both “Bison Pool” and Octopus Spring (Figure 4). In the flow-through experiments, ^13^C-uptake rates into total lipids with acetate was ca. 100 times higher at “Bison Pool” than Octopus Spring, indicating higher turnover and activity of “Bison Pool” streamer communities (Figures 5, 6). However, it should be noted that the experiment at Octopus Spring was shorter than at “Bison Pool,” and may not have been long enough to see similar levels of ^13^C-incorporation. Differences in community composition of SBCs at “Bison Pool” and Octopus Spring might have also accounted for differences in the specific activity of label uptake. Lastly, differences in turnover rates could also be due to differences *in silica* deposition on the cells as higher silica to cell ratio could lead to diffusion limitation to substrates. Carbon assimilation with glucose occurred at both “Bison Pool” and Octopus Spring, underlining the importance of heterotrophic carbon assimilation in SBCs. However, ^13^C-uptake rates into total lipids with glucose were 8 and 26 times lower compared to acetate at Octopus Spring and “Bison Pool,” respectively. Higher uptake rates into lipids with acetate compared to glucose could be explained by the direct utilization of acetate during lipid biosynthesis through formation of acetyl-CoA directly from acetate and Coenzyme-A groups. Glucose on the other hand first undergoes glycolysis before the resulting pyruvate can be converted to acetyl-CoA, which is then used for lipid biosynthesis. Consequently there may be an inherent lag of ^13^C-label uptake into lipids with glucose (and other substrates) relative to acetate. It is also possible that autotrophic organisms can be permeable to acetate (Rittenberg, 1969; Kelly, 1971). Nevertheless, assimilation of organic compounds, including acetate, into biomass is by definition a heterotrophic process. Labeling with ^13^C-formate did not result in significant label incorporation (Table 6; Figure 4), indicating that formate is not a major substrate for SBCs. This is surprising because some thermophilic isolates from Yellowstone National Park hot springs are capable of growing on formate as the sole carbon source (e.g., Huber et al., 1998; Jahnke et al., 2001). It is possible that the flow-through experiments were not conducted long enough to allow induction of growth on formate. However, uptake with formate was also not observed for the batch-fed experiments that were incubated for 1–3 days respectively at “Bison Pool” and Octopus Spring. Instead, ^13^C-label-uptake with formate could be stimulated for archaeal lipids only after the addition of H_2_ at “Bison Pool” (Figure 4, Table 4). This indicates that communities capable of formate assimilation are potentially reductant-limited, and may be energy-limited even at the reducing conditions provided by the hydrothermal fluids of the hot spring.

Despite small isotopic changes for individual lipids in the incubations with ^13^C-bicarbonate, quite large specific carbon uptake rates were observed, owing to the low specific activity of the % ^13^C-label (ca. 1.4% in the flow-through experiments in 2009). Isotopic changes with bicarbonate at “Bison Pool” occurred mainly with saturated C_16_–C_22_ and monounsaturated C_16_ and C_18_ fatty acids (Table 5). There was also carbon assimilation into *i*- and *ai*- branched fatty acids, although accompanied by comparably large errors owing to very small isotopic changes (Figure 5). At Octopus Spring, bicarbonate carbon assimilation was too low to render statistically relevant, as the values were within the range of the analytical error (Table 6, Figure 6). It is also possible that some of the ^13^C-uptake into lipids with bicarbonate could be due to assimilation of inorganic carbon during heterotrophic growth (cf., Werkman and Wood, 1942), which has been shown to account for up to 20% of the lipid carbon of heterotrophic bacteria (Wegener et al., 2012). We therefore cannot exclude the possibility that some of the observed ^13^C-uptake into lipids for ^13^C-bicarbonate could be part of heterotrophic inorganic carbon fixation.

#### ^13^C-uptake into Thermus/Meiothermus and Firmicutes signature lipids

The strongest signs of heterotrophy came from the incorporation of ^13^C-acetate and ^13^C-glucose into branched fatty acids, such as *i*-C_15:0_ and *i*-C_17:0_, which are the most common fatty acids in members of the Thermus/Meiothermus (Yang et al., 2006), Firmicutes (Kaneda, 1991), and some Deltaproteobacteria (Taylor and Parkes, 1983), all of which have been identified as heterotrophic generalists by metagenomic studies conducted at “Bison Pool” (Swingley et al., 2012). This observation is in good agreement with known metabolisms of these organisms as well as their detection in SBCs at “Bison Pool” and Octopus Spring with natural abundance δ^13^C pointing to heterotrophy (Schubotz et al., 2013). Although all of the observed *iso*- and *anteiso* fatty acids could be derived from many different thermophilic organisms (van de Vossenberg et al., 1999), their association to unique head groups such as NAcG-2G-DAG and NAcG-P-DAG distinctly indicates Thermus/Meiothermus as one source organisms. However, according to the IPL data these lipids make up less than 5% of the total community (Figure 3), therefore Thermus/Meiothermus alone cannot account for all the heterotrophic carbon assimilation. Branched C_16_, C_17_, C_18_, and C_19_ fatty acids were also found to be associated to the ubiquitously abundant glycolipids at both “Bison Pool” and Octopus Spring (data not shown) that have an unknown thermophilic bacterial source (Table 2). At “Bison Pool” a large uptake of ^13^C-bicarbonate into *i*-C_17:0_ fatty acid (40 ± 15 ng g^−1^ day^−1^) also indicates that a portion of the Thermus/Meiothermus, Firmicutes (or some as yet unidentified source of *i*-C_17:0_) can also function as autotrophs.

#### ^13^C-uptake into Aquificae signature lipids

Aquificae signature lipids are identified as C_20:1_ and *cy*-C_20:0_ fatty acids and in this study we also included C_20:0_, C_20:2_, C_22:1_, C_22:0_, and *cy*-C_19:0_ fatty acids and C_18:0_, C_19:0_, C_20:1_, C_22:0_ alkanes (derived from bacterial diether lipids by ether cleavage) as Aquificae specific due to their association to APT- and PI-DAGs (data not shown). According to what we know from natural abundance stable carbon isotopes of these fatty acids (Schubotz et al., 2013), we would expect predominantly autotrophic Aquificae at “Bison Pool” (natural abundance δ^13^C of −5 to 5‰), and predominantly heterotrophic Aquificae at Octopus Spring (natural abundance δ^13^C of −27 to 24‰). Indeed, at “Bison Pool,” the sum of carbon uptake into the Aquificae-specific fatty acids is highest with bicarbonate (161 ± 20.6 ng gdw^−1^ day^−1^), followed by acetate (107 ± 0.8 ng gdw^−1^ day^−1^) and negligible with glucose or formate (<5 ng gdw^−1^ day^−1^), while at Octopus Spring there was no uptake detectable with bicarbonate into Aquificae lipids, but also no significant uptake observed with acetate (0.4 ± 0.05 ng gdw^−1^ day^−1^). Notably, carbon uptake into C_18:0_, C_19:0_, C_20:1_, and C_22:0_ alkanes, which are derived from bacterial diether lipids, that are partly assigned to the Aquificae (Table 2) was highest with ^13^C-acetate and glucose at “Bison Pool,” while no significant uptake with ^13^C-formate or bicarbonate was observed. These findings indicate that Aquificae can readily assimilate acetate, glucose and bicarbonate into biomass signifying their mixotrophic nature at “Bison Pool.” Notably, negligible uptake of ^13^C-formate (and ^13^C-glucose into fatty acids) indicates that the Aquificae do not universally metabolize organic compounds. We interpret the failure to detect label-incorporation at Octopus Spring as resulting from lower turnover times for the streamer communities (Table 6) and the fact that flow-through experiments at Octopus Spring were conducted on shorter time-scales compared to Bison Pool.

#### ^13^C-uptake into archaeal lipids

Archaeal community members in biofilm SBCs found at “Bison Pool” and Octopus Spring belong to the order of *Desulfurococcales* and *Thermoproteales* of the Crenarchaea (Meyer-Dombard et al., 2011; Swingley et al., 2012; Schubotz et al., 2013). Many members of these groups are able to grow under autotrophic conditions (Huber et al., 2008; Ramos-Vera et al., 2009), but also heterotrophic growth can occur under both aerobic or anaerobic conditions (Huber and Stetter, 2006; Huber et al., 2006). Natural abundance δ^13^*C*-values for archaeal diether and tetraether lipids are comparably more depleted at Octopus Spring (−26 ± 4‰) than at “Bison Pool” (−14 ± 4‰), indicating a predominantly heterotrophic lifestyle for Archaea at Octopus Spring and an autotrophic or potentially mixotrophic nature at “Bison Pool” (Schubotz et al., 2013). The ^13^C-labeling experiments conducted at both hot springs showed ^13^C-uptake into archaeal lipids with ^13^C-acetate for both batch-fed incubations and flow-through experiments (Figure 4). From this it can be inferred that the SBCs can readily metabolize low molecular weight organic compounds such as acetate. Carbon uptake occurred particularly into archaeol and its ether cleavage product phytane (Tables 3–5), while biphytane 0 (which is derived from GDGT-0) only showed minimal ^13^C-incorporation at Octopus Spring or none at “Bison Pool” in the flow-through experiments and δ^13^*C*-values for biphytanes 1 or 2 remained unaltered. This result can best be understood when considering proposed biosynthetic routes of tetraether lipids, where GDGTs are proposed to form through condensation of two archaeols and subsequent coupling to form the membrane-spanning GDGTs, followed by their subsequent cyclization (Eguchi et al., 2003; Pearson, 2014). We therefore conclude that the biosynthesis of GDGTs was not fast enough to see ^13^C-label incorporation into tetraether lipids in the flow-through experiments. Formate assimilation was only observed at “Bison Pool” when hydrogen was added as supplementary source of reductant (Table 4, Figure 4), indicating that formate can be also utilized by the archaeal communities but only in the presence of additional energy sources. Uptake of ^13^C-glucose, representative of higher molecular weight organic compounds was not significant for archaeal lipids and thus does not seem to be a major carbon source on the timescales of our experiment. Labeling experiments with ^13^C-bicarbonate resulted only in negligible ^13^C-uptake rates into archaeal lipids at “Bison Pool” (<1 ng g^−1^ day^−1^) and none at Octopus Spring. However, ^13^C-bicarbonate uptake was stimulated at “Bison Pool” when hydrogen was added as an additional reductant (Table 4, Figure 4), demonstrating the potential for autotrophic growth of Archaea if additional energy donors are available.

## Summary

This study used a stable isotope labeling approach combined with lipid biomarker analysis to explore modes of carbon assimilation by thermophilic SBCs found in outflow channels of alkaline hot springs. Uptake with ^13^C-acetate into almost all bacterial and archaeal lipids provided direct evidence for a heterotrophic lifestyle of both bacteria and archaea at the two hot springs investigated, “Bison Pool” and Octopus Spring. Heterotrophic carbon assimilation was furthermore confirmed by uptake of ^13^C-glucose into most fatty acids, but specifically *iso* and *anteiso* branched fatty acids, which are specific to the Thermus/Meiothermus and Firmicutes. Significant ^13^C-bicarbonate uptake into bacterial fatty acids occurred only at “Bison Pool” and mainly into saturated and monounsaturated fatty acids and some Aquificae-diagnostic fatty acids, but not at Octopus Spring. Formate could not be confirmed as a major substrate for SBCs, but growth with formate could be stimulated by the addition of hydrogen as an extra electron donor.

Our results confirm observations of natural stable carbon isotopes of lipids at Octopus Spring indicating that most of the SBCs are heterotrophic, and “Bison Pool” where δ^13^*C*-values have pointed to the presence of mixotrophic communities. We calculate through carbon uptake rates that microbial communities at “Bison Pool” were 100 times more active than at Octopus Spring, indicating that SBCs at Octopus might be comparably growth-limited.

### Conflict of interest statement

The authors declare that the research was conducted in the absence of any commercial or financial relationships that could be construed as a potential conflict of interest.
